# Responsiveness of the Japanese version of the Pregnancy‐Related Anxiety Questionnaire‐Revised 2 and its predictive validity for postnatal maternal mental health: A longitudinal study in Japan

**DOI:** 10.1111/jog.70085

**Published:** 2025-09-18

**Authors:** Ritsuko Shirabe, Hiroko Okada, Tsuyoshi Okuhara, Takahiro Kiuchi

**Affiliations:** ^1^ University Hospital Medical Information Network (UMIN) Center, the University of Tokyo Hospital Tokyo Japan; ^2^ Department of Health Communication, School of Public Health, Graduate School of Medicine The University of Tokyo Tokyo Japan

**Keywords:** anxiety, depression, health communication, pregnancy, psychiatric status rating scales

## Abstract

**Aim:**

The extent to which pregnancy‐related anxiety predicts postnatal maternal mental health remains unclear. This study aimed to evaluate the responsiveness and predictive validity of the Japanese version of the Pregnancy‐Related Anxiety Questionnaire‐Revised 2 in identifying women at high risk for postnatal mental health problems.

**Methods:**

A longitudinal study recruited 218 Japanese women in early pregnancy from three facilities and followed them through the postpartum period. Participants completed the scale three times (in early, mid‐, and late pregnancy). Responsiveness was analyzed using one‐way repeated measures Analysis of Variance. At 1 month postpartum, the Parenting Stress Index and the Edinburgh Postnatal Depression Scale assessed parenting stress and depression. Spearman's correlations were calculated between the scale and parenting stress, and Receiver Operating Characteristic curves examined its predictive validity for depression.

**Results:**

A total of 211 women were followed up. The scale scores were highest in early pregnancy (*p* < 0.001). Spearman's correlation coefficients between pregnancy‐related anxiety and postnatal parenting stress ranged from 0.34 to 0.43 across the pregnancy stages. Areas under the curve for predicting postnatal depression were 0.74 (early), 0.79 (mid‐), and 0.77 (late pregnancy). Based on the set threshold, women with high scale scores had significantly increased relative risks (95% confidence intervals) of postnatal depression: 3.4 (1.4–8.2) (early), 5.2 (2.5–10.7) (mid‐), and 6.9 (2.7–18.0) (late pregnancy).

**Conclusions:**

This scale showed good responsiveness and predictive validity, making it a valuable tool for identifying women at risk of postnatal mental health issues and evaluating intervention efficacy in clinical settings.

## INTRODUCTION

Postpartum depression, as well as other maternal mental health problems during the postpartum period, is a serious worldwide issue: the global pooled prevalence of postpartum depression is as high as 18%.^1^ Maternal mental health is particularly vulnerable to deterioration during the first month postpartum; for example, depression may occur in response to adapting to life with a newborn.^2^ Postpartum depression can be predicted based on mood disorders during pregnancy^3^, ^4^; therefore, emotional support should be initiated during the prenatal period to prevent deterioration in maternal mental health after childbirth.

The most common mood disorder during the perinatal period is anxiety, with a prevalence rate of 15% for anxiety disorders and 23% for anxiety symptoms.^5^ Pregnancy‐related anxiety is defined as nervousness and fear about the following: the baby's health; the mother's health and appearance; the experience with the healthcare system; and social and financial issues in the context of pregnancy, childbirth, and parenting.^6^ Pregnancy‐related anxiety is associated with postpartum maternal parenting stress because of its sensitivity in capturing individual vulnerability to stressors and anxieties related to specific, temporary demands that arise during pregnancy.^7^, ^8^ It fluctuates during pregnancy among individuals: previous studies have reported a downward trajectory^9^ and a U pattern (high levels of anxiety were observed in early and late pregnancy).^10^, ^11^ When pregnancy‐related anxiety is viewed as an emotional stress response, it may theoretically lead to a subsequent decline in mental health.^12^ In fact, it has been reported that pregnancy‐related anxiety more strongly predicts postpartum affective disturbances such as parenting stress and anxiety disorders than general anxiety.^8^, ^13^

Among existing scales which can measure multifaceted pregnancy‐related anxiety, the Pregnancy‐Related Anxiety Questionnaire‐Revised 2 (PRAQ‐R2) can be completed by women with or without previous childbirth and assess prenatal anxiety about child health, child loss, childbirth, and body image.^10^ This scale (including the previous version) has been reported to predict various adverse outcomes such as increased medical intervention^13^ and postpartum parenting stress.^8^ We previously developed the Japanese version of the PRAQ‐R2 and examined its psychometric properties.^14^ This instrument is reliable and able to capture the intricacies of multifaceted pregnancy‐related anxiety among Japanese pregnant women; however, because of its cross‐sectional design, we could not evaluate the responsiveness (intrapersonal changes) or predictive validity of the scale for postpartum maternal mental health. Although it makes sense in theory, it is still unclear to what extent pregnancy‐related anxiety can actually predict severe maternal stress and depression during the early postpartum period.

Using a longitudinal design, this study aimed to confirm the responsiveness and the predictive validity of the Japanese version of the PRAQ‐R2. We hypothesized that this scale captures the intrapersonal changes in pregnancy‐related anxiety scores during pregnancy, and that it can serve as a screening tool for the deterioration of postnatal maternal mental health.

## METHODS

### Settings and participants

We conducted this study as a part of a longitudinal study that followed women's emotional responses from early pregnancy through the postpartum period. The present study took place at one facility in Tokyo (a regional perinatal medical center) and two clinics in Kanagawa. Eligible participants were outpatients who met the following criteria: (1) Japanese; (2) aged 18 years or older; (3) pregnant with one baby; (4) in early pregnancy (<15 weeks); (5) had no problems reading Japanese; and (6) were planning to deliver at one of the three study facilities. Participants were recruited through consecutive sampling orally and in writing during regular checkups by the first author or other research collaborators at each facility between April 2022 and May 2023. They were informed that participation was voluntary and that they could return the questionnaires either by hand or by mail. For the longitudinal study, participants answered questionnaires at five time points: early pregnancy (< 15 weeks: T1), mid‐pregnancy (15–27 weeks: T2), late pregnancy (>27 weeks: T3), in hospital after childbirth (T4), and 1 month postpartum (T5). For the current study, we used the questionnaires completed at four time points (all except T4). Signed consent was obtained from the participants before they were given the first questionnaire. We sent participants a 1000‐yen (roughly equivalent to 7 USD during the study period) gift certificate following receipt of the last questionnaire. This study was approved by the Institutional Review Board of the University of Tokyo (approval code: 2020154NI).

### Measures

#### Pregnancy‐related anxiety

We used the Japanese version of the PRAQ‐R2, which is a reliable and validated scale for assessing pregnancy‐related anxiety^14^ and developed by referring to the original scale.^10^ The PRAQ‐R2 is a 10‐item self‐report measure with three factors: “Fear of giving birth”, “Worries about bearing a handicapped child”, and “Concern about own appearance”. Items are rated from 1 (absolutely not relevant) to 5 (very relevant), with higher total scores indicating stronger anxiety.

In this study, omega (total) values^15^ for the overall scale at each time point were 0.90 (T1), 0.90 (T2), and 0.93 (T3), indicating good internal consistency. Omega (total) values for the three factors were as follows: fear of giving birth, 0.79 (T1), 0.78 (T2), and 0.81 (T3); worries about bearing a handicapped child, 0.94 (T1), 0.92 (T2), and 0.94 (T3); and concern about own appearance, 0.77 (T1), 0.79 (T2), and 0.84 (T3).

#### Parenting stress

To examine the predictive validity of the Japanese version of the PRAQ‐R2, we assessed parenting stress with the Japanese version of the Parenting Stress Index,^16^ which was developed for Japanese mothers by referring to the original scale.^17^ In accordance with a previous study,^8^ we used only the parenting domain (containing 40 items rated on a five‐point Likert scale) because the child‐specific domain could not be applied at 1 month postpartum. The parenting domain (omega (total) value = 0.93) consists of eight subscales: role restriction (seven items; omega = 0.88); isolation (seven items; omega = 0.86); spouse (five items; omega = 0.88); competence (seven items; omega = 0.87); depression (four items; omega = 0.89); sad/uneasy feeling after leaving the hospital (four items; omega = 0.82); attachment (three items; omega = 0.70); and health (three items; omega = 0.70). The items where a high score represented low parenting stress were reverse scored, so that higher sums of all items indicated high parenting stress.

#### Depression

Depressive symptoms were assessed at two time points (T1 and T5), using the Japanese version of the Edinburgh Postnatal Depression Scale (EPDS),^18^ developed by referring to the original scale that can assess perinatal depressive states.^19^ This is a validated and reliable 10‐item self‐report measure, and each item is rated on a four‐point Likert scale ranging from 0 to 3. Participants were identified as having depression using a score of 9 or more out of 30, based on a previous study among Japanese women.^18^ The omega (total) values for the EPDS in this study were 0.88 (T1) and 0.90 (T5).

#### Obstetric, psychological, and social characteristics

In the T1 questionnaire, participants were also asked questions about their socioeconomic status (annual income and educational background) and a history of abuse and/or domestic violence. Social support was evaluated using the Japanese version of the Social Support Questionnaire (J‐SSQ)^20^ with reference to the original scale.^21^ The J‐SSQ comprises 12 items and has two factors: number of persons (NP) and satisfaction rating (SR). NP is the sum of the perceived numbers of available others in six different situations. SR is the sum of the degree of satisfaction in these six situations, ranging from 1 (very dissatisfied) to 6 (very satisfied). Its reliability and validity during pregnancy have been confirmed.^22^ The overall omega total and two factors (NP and SR) of the J‐SSQ were 0.90, 0.92, and 0.98, respectively.

Participants' age, number of past deliveries and miscarriages, means of conception, height and weight before pregnancy, and whether they had any high‐risk medical conditions or any history of mental illness before or during pregnancy were obtained from medical records.

### Statistical analysis

To confirm the responsiveness of the PRAQ‐R2, the means for pregnancy‐related anxiety at T1, T2, and T3 were compared using a one‐way repeated measures Analysis of Variance (ANOVA). We assessed pairwise comparisons using Tukey's test for multiple comparisons.

To confirm the predictive validity of the PRAQ‐R2, we evaluated the correlations between pregnancy‐related anxiety in each trimester and postnatal parenting stress by drawing distribution charts and then calculating Spearman correlation coefficients (ρ). The correlation strength is classified according to Evans's criteria as follows: 0.00–0.19 (very weak), 0.20–0.39 (weak), 0.40–0.59 (moderate), 0.60–0.79 (strong), and 0.80–1.00 (very strong).^23^ To confirm its predictive validity for postpartum depression, we performed a multivariate logistic regression analysis to examine the association between PRAQ‐R2 scores and postpartum depression, controlling for risk factors for postpartum depression reported in previous systematic reviews.^24^, ^25^ In addition to age (continuous) and previous deliveries (number), we chose factors measured prior to exposure (i.e., pregnancy‐related anxiety) as follows: history of depression (yes/no), having depression at T1 (yes/no), history of abuse and/or domestic violence (yes/no), body mass index (BMI) before pregnancy (<18.5, 18.5–<25.0, ≥25.0: the categories with different weight gain guidance policies in Japan), social support (NP) (continuous), social support (SR) (continuous), and gestational diabetes diagnosed during pregnancy (yes/no, only entered in an analysis of T3 score). Next, we drew receiver operating characteristic (ROC) curves between pregnancy‐related anxiety in each trimester and postnatal depression. Discriminatory ability was evaluated by area under the curve (AUC). After identifying the optimal threshold at the point closest to the top‐left part of each ROC curve, we determined a cut‐off point that can be used commonly throughout pregnancy for clinical use by calculating sensitivity and specificity at each point. We calculated relative risks for postnatal depression in each trimester using the determined threshold.

Most of the missing values were unit non‐responses (unanswered questionnaires at one time point). With the exception of those participants who dropped out, it is likely that research collaborators failed to distribute questionnaires because of the busy work environment at each facility; such values were assumed to be missing at random. An available case analysis (i.e., a complete case analysis for each analysis) was performed for missing values. The threshold for significance was set at *p* < 0.05. All statistical analyses and figures were done using R for Windows (version 4.0.2, R Foundation for Statistical Computing, Vienna, Austria).

## RESULTS

We recruited a total of 218 participants. After recruitment (but prior to T1), seven women dropped out: four were transferred to different hospitals, two were hospitalized, and one withdrew because of intense anxiety. Table 1 shows the demographic and obstetric characteristics of the participants at baseline (T1). Past mental illnesses included maladjustment and/or panic disorder (*n* = 9), depression (*n* = 5), and insomnia (*n* = 1). There was an overlap between these conditions.

**TABLE 1:** Participant characteristics (*n* = 211).

Variables	*n*	%
Age (years), median (IQR^a^)	32 (31–36)	
Previous delivery (number)		
0	86	41
1	102	48
2	20	9.5
3	3	1.4
Previous abortion		
Yes	57	27
Current/past complications		
Yes	58	27
Means of conception		
Natural	186	88
Artificial insemination	3	1.4
In vitro fertilization	21	10
Syringe (donor sperm)	1	0.5
History of mental illness		
Yes	14	7
Depressive states at early pregnancy		
Yes	23	11
Education		
≤15 years	16	8
17 years	46	22
19 years	120	57
≥21 years	14	7
Having a paid job	164	78
Full‐time	151	72
Annual household income (JPY^b^)		
<4.0 million	16	8
4.0–5.9 million	23	11
6.0–7.9 million	32	15
8.0–10 million	48	23
>10 million	75	36
Body mass index before pregnancy (kg/m^2^)		
<18.5	33	16
18.5–<25.0	162	77
≥25.0	16	8
Marital status		
Non‐single (including prospective and de facto marriages)	210	99.5
History of abuse and/or domestic violence		
Yes	10	5
Social support		
Number of persons, median (IQR^a^)	22 (17–30)	
Satisfaction rating, median (IQR^a^)	30 (29–36)	
Gestational diabetes at late pregnancy (T3)		
Yes	9	

^a^ Interquartile range.

^b^ Roughly equivalent to USD 0.007 during the study period.

Appendices I shows a flow chart of participants after recruitment. In summary, a total of 39 women dropped out during the study period. Participants who dropped out were slightly older (mean = 34.2 vs. 32.7 years, *p* = 0.047), had experienced a higher number of abortions (mean = 0.91 vs. 0.26 times, *p* < 0.001), and were in lower income categories (*p* = 0.030) than women followed through T5. For the other characteristics (including parity, means of conception, educational background, past/current complications at baseline, history of mental illness, and having a job, as well as baseline pregnancy‐related anxiety scores), there were no differences between the women who dropped out and those who completed the study. Participants answered each questionnaire at mean (standard deviation: SD) gestational weeks: 9.6 (1.7) at T1, 24 (1.2) at T2, and 31 (1.7) at T3.

### Responsiveness of the Japanese version of the PRAQ‐R2

**FIGURE 1:**
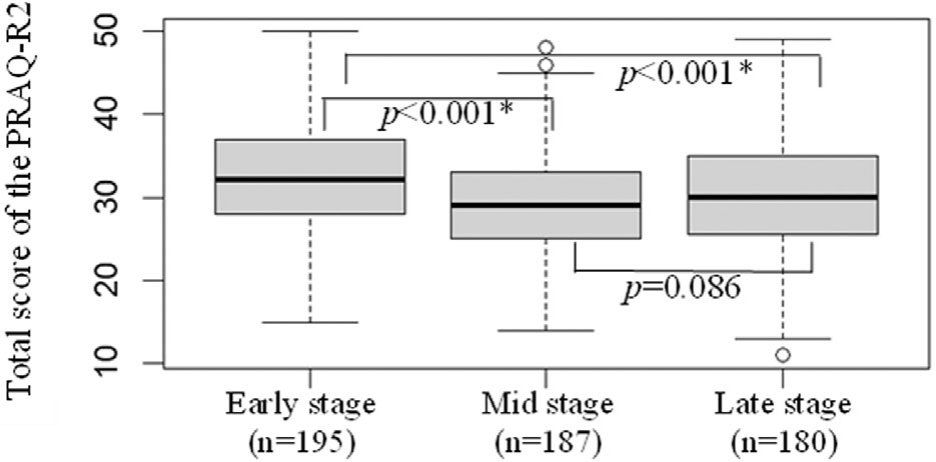
Distribution of the pregnancy‐related anxiety scores across the three trimesters of pregnancy as assessed by the Japanese version of the PRAQ‐R2. *p < 0.05.

Figure 1 presents the boxplot illustrating the total scores of the Japanese version of the PRAQ‐R2 across the three trimesters of pregnancy. The mean (SD) total score in the early stage of pregnancy was 32.4 (6.8), which was significantly higher than the scores observed in the middle (29.5 (6.5)) and late stages of pregnancy (30.2 (7.0)).

Appendices II shows the changes in scores for each subscale of the PRAQ‐R2 during pregnancy. Among the subscales, the scores for fear of childbirth declined from early pregnancy (mean (SD) = 9.7 (2.6)) to mid‐pregnancy (mean (SD) = 8.6 (2.5)), but increased again in late pregnancy (mean (SD) = 9.3 (2.6)). Similarly, the scores for worries about bearing a handicapped child showed a decrease from early pregnancy (mean (SD) = 14.2 (3.8)) to mid‐pregnancy (mean (SD) = 12.1 (3.4)).

### Predictive validity of the Japanese version of the PRAQ‐R2

**FIGURE 2:**
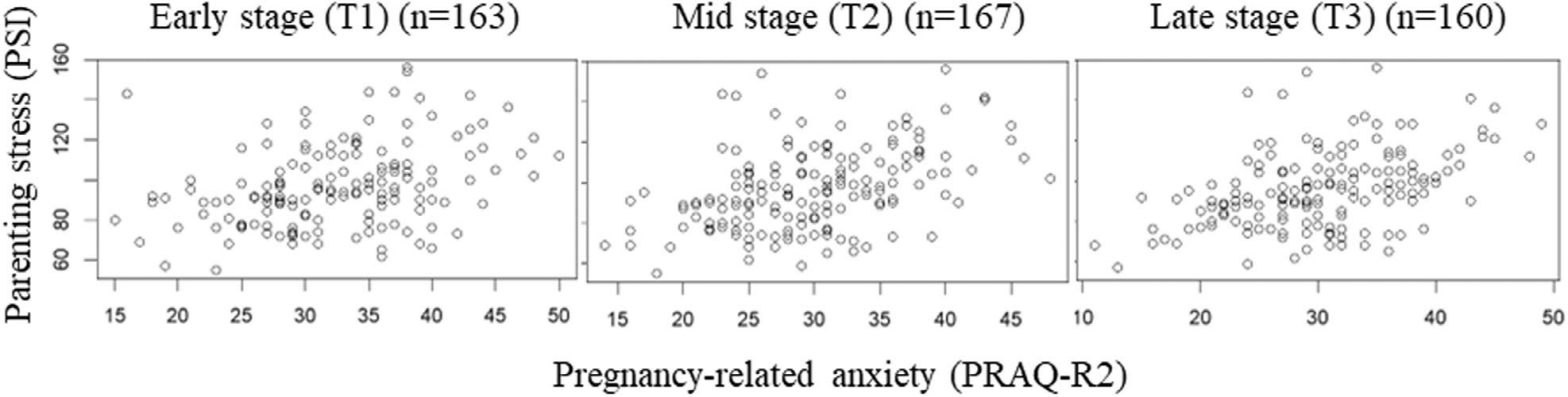
Distribution charts between the scores on the Japanese version of the PRAQ‐R2 in the three trimesters of pregnancy and the scores on the Japanese version of the Parenting Stress Index at 1 month postpartum.

Figure 2 shows the distribution charts between pregnancy‐related anxiety in each trimester and postnatal parenting stress. Spearman correlation coefficients (ρ) were 0.34 (T1), 0.43 (T2), and 0.43 (T3), respectively.

Among the participants who answered the EPDS questionnaire at T5 (*n* = 176), 26 (15%) women were identified as having depression. Table 2 presents the results of the multiple logistic regression analysis that predicts depressive symptoms at postpartum 1 month. Even after adjusting for risk factors for postpartum depression, the odds ratio increased with each 1‐point increase in the PRAQ‐R2 score, indicating that pregnancy‐related anxiety was positively associated with postpartum depression at all gestational stages. Mean scores of the PRAQ‐R2 in the postpartum depression group were 37.3 (T1), 35.8 (T2), and 36.5 (T3), which were higher than the non‐depression group: 31.6 (T1), 28.6 (T2), and 29.3 (T3).

**TABLE 2:** Multivariate logistic regression analysis predicting postnatal depressive state.

Variable	Early stage (T1, *N* = 154)		Mid‐stage (T2, *N* = 151)		Late‐stage (T3, *N* = 144)	
	OR^a^	95% CI^b^	OR^a^	95% CI^b^	OR^a^	95% CI
Pregnancy‐related anxiety	1.01	1.00–1.02	1.02	1.01–1.03	1.02	1.01–1.02
Age	1.00	0.99–1.02	1.00	0.99–1.02	0.996	0.98–1.01
Previous deliveries	0.96	0.89–1.04	0.99	0.91–1.07	0.98	0.91–1.06
History of depression	0.83	0.60–1.15	0.81	0.59–1.12	0.80	0.59–1.09
Depressive state at T1	1.32	1.12–1.56	1.26	1.07–1.50	1.30	1.11–1.53
History of abuse or domestic violence	1.35	1.02–1.79	1.33	1.01–1.76	1.30	0.98–1.73
BMI^c^ before pregnancy						
<18.5	1.11	0.95–1.28	1.01	0.87–1.18	1.00	0.87–1.16
≥25.0	1.06	0.85–1.33	1.04	0.83–1.30	1.05	0.85–1.30
Social support						
Number of persons^d^	1.02	0.91–1.14	1.04	0.93–1.16	1.01	0.90–1.13
Satisfaction rating	1.01	0.998–1.01	1.01	0.998–1.01	1.00	0.999–1.01
Gestational diabetes	—	—	—	—	1.11	0.88–1.40

^a^ Odds ratio.

^b^ Confidence interval.

^c^ Body mass index.

^d^ Log transferred.

**FIGURE 3:**
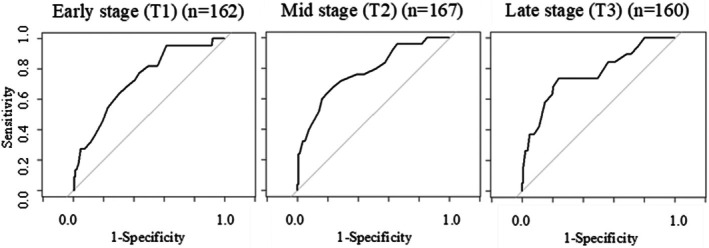
ROC curves between the scores on the Japanese version of the PRAQ‐R2 in the three trimesters of pregnancy and a positive postnatal depression screening on the EPDS.

Figure 3 shows the ROC curves of pregnancy‐related anxiety score in each trimester when predicting postnatal depression. AUC was 0.74 (T1), 0.79 (T2), and 0.77 (T3), respectively. The points closest to the top‐left part of the plot in the ROC curves were 35.5 (sensitivity = 0.70, specificity = 0.65) at T1, 32.5 (sensitivity = 0.76, specificity = 0.69) at T2, and 33.5 (sensitivity = 0.74, specificity = 0.76) at T3. Table 3 shows the sensitivity and specificity at each stage of pregnancy when each score is used as the cutoff value. Based on these results, we set the threshold of low/high pregnancy‐related anxiety as 33/34 for this study. Those women who had a PRAQ‐R2 score of 34 or more were more likely to test positive for depression when screened at 1 month postpartum than those who had lower scores in all trimesters: the relative risks (95% confidence intervals) were 3.4 (1.4–8.2) at T1, 5.2 (2.5–10.7) at T2, and 6.9 (2.7–18.0) at T3, respectively.

**TABLE 3:** Sensitivity and specificity for each cut‐off value of the Pregnancy–Related Anxiety Questionnaire‐Revised 2 in predicting postnatal depression.

Cut‐off score	Early stage (T1)		Mid‐stage (T2)		Late stage (T3)	
	Sensitivity	Specificity	Sensitivity	Specificity	Sensitivity	Specificity
32	0.83	0.51	0.73	0.71	0.75	0.63
33	0.78	0.56	0.69	0.76	0.75	0.70
34	0.74	0.59	0.65	0.80	0.75	0.76
35	0.70	0.65	0.62	0.84	0.70	0.80
36	0.65	0.70	0.54	0.86	0.60	0.80

## DISCUSSION

This study examined intrapersonal changes in pregnancy‐related anxiety throughout pregnancy as assessed by the previously developed Japanese version of the PRAQ‐R2. Total PRAQ‐R2 scores were highest in the early stage of pregnancy. This longitudinal study also investigated whether pregnancy‐related anxiety, when assessed during pregnancy, could predict postnatal maternal mental health. Pregnancy‐related anxiety was correlated with postnatal parenting stress and predicted a postnatal depressive state.

Given that pregnancy‐related anxiety fluctuates during pregnancy, this study assessed the responsiveness of the Japanese version of the PRAQ‐R2 for the first time. In line with a previous study, this study found that pregnancy‐related anxiety scores were highest in the early stage of pregnancy across the three trimesters.^9^ In the multifaceted pregnancy‐related anxiety, fear of childbirth is reported to increase as birth approaches.^10^, ^11^ Consistent with those reports, in this study the “fear of childbirth” subscale showed a U‐pattern change across the three trimesters and increased from mid to late pregnancy. Thus, the Japanese version of the PRAQ‐R2 can capture the fluctuation of pregnancy‐related anxiety during pregnancy, supporting its good responsiveness.

In the present study, pregnancy‐related anxiety in mid‐ and late pregnancy was moderately correlated with postpartum parenting stress. A previous study reported correlation coefficients between pregnancy‐related anxiety in the second and third trimesters and parenting stress at 3 months postpartum as 0.28–0.46,^8^ which were generally consistent with those of the present study. That study also found that neither prenatal depression nor state anxiety predicted a high level of parenting stress when tested for simultaneously with pregnancy‐specific anxiety.^8^ Our study suggests that women with high pregnancy‐related anxiety levels in mid‐ to late pregnancy require special care through the postpartum period because they may be particularly vulnerable to transitional stressors.

In the present study, pregnancy‐related anxiety was associated with postpartum depression at all stages of pregnancy even after adjusting for antenatal risk factors for postpartum depression. ROC curves between pregnancy‐related anxiety and postnatal depression showed sufficient AUC values in all trimesters, supporting this scale's reasonable accuracy in predicting a depressive state at 1 month postpartum.^26^ Theoretically, pregnancy‐related anxiety can be perceived as an emotional response to a stressor.^12^ Stress hormones activate the hypothalamic–pituitary–adrenal axis, causing an increase in corticotropin‐releasing hormone, and clinical studies have shown that this is frequently associated with postpartum depression.^27^ Pregnancy‐related anxiety has been reported to be strongly associated with maternal blood cortisol levels,^28^ and such physiological mechanisms and psychosocial factors may be involved in predicting postpartum depression.^27^

We established a single threshold to predict postpartum depression that can be used throughout pregnancy, balancing sensitivity and specificity with clinical utility. In this process, the cutoff value for early pregnancy was lowered from that calculated from the ROC curve, resulting in increased sensitivity and decreased specificity in early pregnancy. The rationale for increasing sensitivity in this context was based on the consideration that screening using this scale would not be performed for the purpose of additional detailed examinations or aggressive treatment, but rather to ensure that women with moderate or severe anxiety are not overlooked and receive care from early pregnancy onward.^29^ Based on the threshold, the relative risks for postpartum depression among women with high pregnancy‐related anxiety scores increased threefold to sevenfold from the early to late stages of pregnancy.

The PRAQ‐R2 can capture pregnancy‐specific anxiety that differs from general anxiety.^14^, ^30^ This scale can identify women at a high risk for postpartum strong stress and depressive states who would not have been identified by general anxiety measures; therefore, it will enable targeted prenatal care to be initiated for these women in the early stages of pregnancy. It has been suggested that increased anxiety during pregnancy may be caused by insufficient interaction between healthcare providers and women, as well as a lack of communication.^31^ By using this scale, it is possible to share the pregnancy‐specific anxiety with women who tested positive in the screening and consider the necessary support for them. In addition, pregnant women who experience severe anxiety until the late stages of pregnancy are at high risk of deteriorating mental health after childbirth. Therefore, by tracking anxiety scores until the late stages of pregnancy, it is possible to verify the effectiveness of support during pregnancy and provide continuous support until after childbirth to the high‐risk group identified in the late stages. Future research is needed to explore what kind of support can alleviate overall or specific types of pregnancy‐related anxiety.

This study has several limitations. First, although we recruited participants at multiple facilities, there may have been selection bias. Our data on age, marital status, and means of conception were similar to those reported in Japanese national data; however, socioeconomic backgrounds were rather homogenous, possibly because this study was conducted in an urban area. Future studies will need to target women who have lower socioeconomic backgrounds. Second, in dealing with missing values which were not completely at random, available case analysis may have affected the results of this study. Third, in many cases, the reasons why participants dropped out during the study are unknown (missing without having quit). Although their baseline pregnancy‐related anxiety scores were not different from those of women who were followed through to the postpartum period, women who were unable to continue participating in this study may have exhibited unique anxiety fluctuations or effects on their postpartum mental health. Despite these limitations, this study is the first to show variation in pregnancy‐related anxiety assessed by the Japanese version of the PRAQ‐R2 from early to late pregnancy. In addition, this is the first study to demonstrate that pregnancy‐specific anxiety can predict postnatal depression, which had previously only been theorized.

In conclusion, the Japanese version of the PRAQ‐R2 has good responsiveness and can capture intrapersonal changes in anxiety levels during pregnancy. Future studies, including intervention programs, can use this scale to test for effectiveness in reducing pregnancy‐specific anxiety. We also found that this scale can be used to predict some degree of postpartum maternal parenting stress and depression from pregnancy‐related anxiety experienced in the prenatal period. By utilizing this scale to identify pregnant women with high levels of pregnancy‐related anxiety who do not fit the traditional definition of anxiety disorders, professionals can provide early care and intervention during pregnancy, as well as long‐term follow‐up care throughout the postpartum period.

## AUTHOR CONTRIBUTIONS

**Ritsuko Shirabe:** Conceptualization; methodology; validation; software; formal analysis; data curation; funding acquisition; investigation; writing – original draft; project administration; resources; visualization. **Hiroko Okada:** Conceptualization; methodology; funding acquisition; writing – review and editing; validation; investigation. **Tsuyoshi Okuhara:** Visualization; writing – review and editing; methodology; conceptualization; project administration; validation. **Takahiro Kiuchi:** Conceptualization; supervision; resources; writing – review and editing; project administration; methodology.

## CONFLICT OF INTEREST STATEMENT

The authors declare no conflicts of interest.

## ETHICS STATEMENT

This study was approved by the Institutional Review Board of the University of Tokyo (approval code: 2020154NI).

## INFORMED CONSENT STATEMENT

Signed consent was obtained from the participants.

## Supporting information


**Appendix I.** Participants flow chart.

**Appendix II.** Distribution of the scores of each subscale in the Japanese version of the PRAQ‐R2 across three trimesters of pregnancy. *p < 0.05.

## Data Availability

This study used explanatory documents approved by the Institutional Review Board of the University of Tokyo. Participants have given their consent after being informed that their personal data will not be disclosed to outside parties and that only the results of the study will be published in academic journals; therefore, personal data from this study is not publicly available. However, the data that support the findings of this study are available on request from the corresponding author with an explanation of the above handling procedures.
