# Supplementary figures and images for: Responsiveness of the Japanese version of the Pregnancy‐Related Anxiety Questionnaire‐Revised 2 and its predictive validity for postnatal maternal mental health: A longitudinal study in Japan
Source: J Obstet Gynaecol Res. 2025 Sep 18;51(9):e70085. doi: 10.1111/jog.70085 (PMC12446746; doi:10.1111/jog.70085)

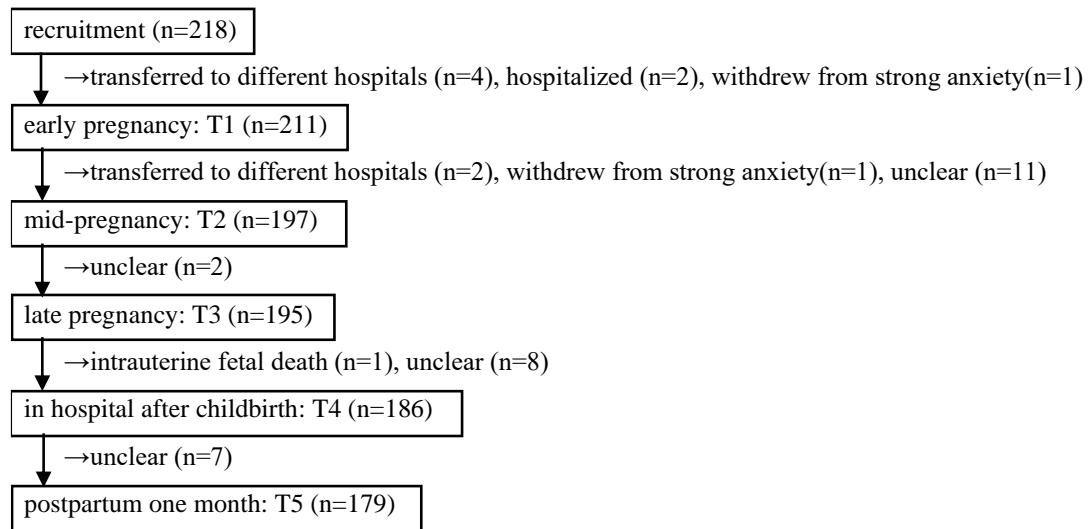

Supplement: Supplementary file 1 — Appendix I. Participants flow chart. [file JOG-51-0-s001.pdf]

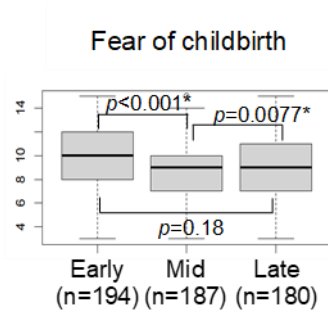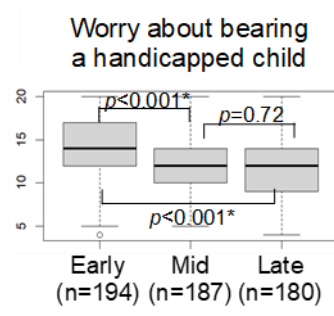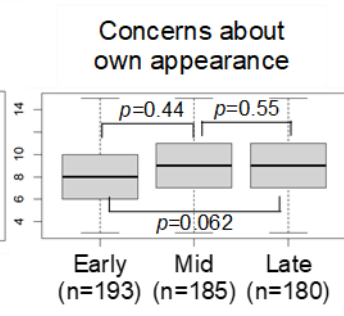

Supplement: Supplementary file 2 — Appendix II. Distribution of the scores of each subscale in the Japanese version of the PRAQ‐R2 across three trimesters of pregnancy. *p < 0.05. [file JOG-51-0-s002.pdf]
